# Redox Homeostasis and Immune Alterations in Coronavirus Disease-19

**DOI:** 10.3390/biology11020159

**Published:** 2022-01-19

**Authors:** Francesco Bellanti, Aurelio Lo Buglio, Gianluigi Vendemiale

**Affiliations:** Department of Medical and Surgical Sciences, University of Foggia, Viale Pinto 1, 71122 Foggia, Italy; aurelio.lobuglio@unifg.it (A.L.B.); gianluigi.vendemiale@unifg.it (G.V.)

**Keywords:** COVID-19, redox balance, immune response

## Abstract

**Simple Summary:**

Severe Acute Respiratory Syndrome Coronavirus 2 (SARS-CoV-2) has caused the Coronavirus Disease 2019 (COVID-19) pandemic, which may present with a wide clinical presentation. The capability of preventing serious illness with early interventions or managing severe disease is of extreme importance, encouraging the search for therapeutic targets. We review current evidence on the involvement of oxidant molecules with severe infection and lung injury in COVID-19. Reactive species and redox imbalance may dysregulate the immune response and account for disease progression in SARS-CoV-2 infection. This aspect suggests treatment options that could hinder disease progression and prevent multiple features of severe illness, which include clotting predisposition, cytokine storm and organ damage.

**Abstract:**

The global Coronavirus Disease 2019 (COVID-19) pandemic is characterized by a wide variety of clinical features, from no or moderate symptoms to severe illness. COVID-19 is caused by the Severe Acute Respiratory Syndrome Coronavirus 2 (SARS-CoV-2) that first affects the respiratory tract. Other than being limited to lungs, SARS-CoV-2 may lead to a multisystem disease that can even be durable (long COVID). The clinical spectrum of COVID-19 depends on variability in the immune regulation. Indeed, disease progression is consequent to failure in the immune regulation, characterized by an intensification of the pro-inflammatory response. Disturbance of systemic and organ-related redox balance may be a further mechanism underlying variability in COVID-19 severity. Other than being determinant for SARS-CoV-2 entry and fusion to the host cell, reactive species and redox signaling are deeply involved in the immune response. This review sums up the present knowledge on the role of redox balance in the regulation of susceptibility to SARS-CoV-2 infection and related immune response, debating the effectiveness of antioxidant compounds in the management of COVID-19.

## 1. Introduction

The Coronavirus Disease 2019 (COVID-19) was declared a worldwide pandemic by the World Health Organization (WHO) on 11 March 2020 [1]. After the first diagnosis of COVID-19 performed in Wuhan (China) in December 2019, the disease spread rapidly, and it now affects 222 territories and countries [2]. Severe Acute Respiratory Syndrome Coronavirus 2 (SARS-CoV-2) is the etiologic agent of COVID-19. Even though the origin of SARS-CoV-2 opens a burning debate, it most likely derives from natural selection in an animal host succeeded by zoonotic transfer [3]. Elements of SARS-CoV-2 infectivity and transmissibility, together with various clinical manifestations of COVID-19, represent hot research topics, particularly with the worrying spread of different variants. Acute respiratory failure is the most frequent presentation of severe COVID-19, but various non-respiratory clinical conditions may be included in both the acute illness and the post-COVID syndrome (or long COVID) [4].

Severe COVID-19 is most often reported in elderly patients with comorbidities, while young people commonly present with mild disease [5,6]. The different age-related course of disease may be dependent on variations in the immune response, since the immune system undertakes a complex process of maturation from birth to adult age, and aging is related to several immune modifications [7]. Indeed, elderly patients present with a lower capability to react to viral infections and a higher baseline pro-inflammatory state than young or adult subjects [8,9]. Immune response to viruses includes both innate and adaptive immune systems. Briefly, innate mechanisms involve virus identification by the toll-like receptors (TLR) of macrophages and dendritic cells (DC), which in turn produce type I interferon (IFN) and pro-inflammatory cytokines, such as interleukin (IL)-1β, IL-6, tumor necrosis factor (TNF) and chemokines, leading to the recruitment of neutrophils and further inflammatory immune cells to the site of infection. The adaptive immune response is triggered by viral antigen presentation to CD4 helper T (Th) cells and CD8 cytotoxic T cells: while the latter kill viral infected cells, Th cells activate B cells to release neutralizing antibodies. Even though such immune response mediates protective immunity, excessive and improper release of pro-inflammatory cytokines may lead to cytokine storm, with consequent clinical complications and death [10].

Changes in redox balance are determinant for the immunity and inflammation. On one hand, redox reactions modulate the immune response, regulating the spatial and temporal immunological processes [11]. On the other hand, activated immune cells rearrange their redox system to trigger cytocidal reactions within the pathogen defense strategy [12]. Alterations in redox balance described by excess in reactive species overwhelming antioxidant defense may lead to oxidative stress, which is characteristic of several viral infections [13]. Thus, redox disbalance and immune response are intertwined processes that may play a crucial role in COVID-19 progression and response to therapy via interference with several signaling pathways.

After a brief presentation of redox homeostasis and of the immune response in SARS-CoV-2 infection, the present review presents the latest evidence on possible interconnections between dysregulation of redox balance and immune alterations in COVID-19.

## 2. Redox Homeostasis: Reactive Species and Antioxidants

### 2.1. Redox Biology and Oxidative Stress

**Figure 1:**
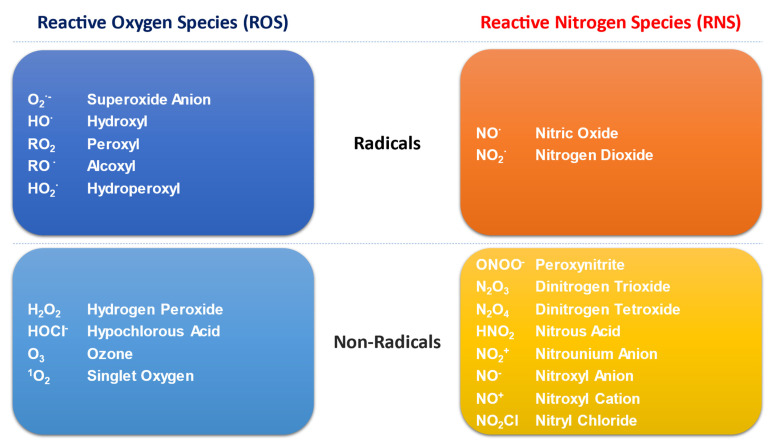
Schematic representation of different types of oxidants-or reactive species-produced by cell metabolism.

Major metabolic pathways and their breakdown products are required to meet the cellular energetic and synthetic demands, including the responses of immune cells. Metabolic reactions produce both reactive oxygen and nitrogen species (ROS and RNS, respectively), globally termed as reactive species or oxidants (Figure 1). Such molecules are classified as free radicals (with one or more unpaired electrons in their outer shell) and non-radical compounds. Free radicals comprise superoxide anion (O_2_·^−^) hydroxyl radical (HO·), nitric oxide (NO·) and nitrogen dioxide (NO_2_·), while hydrogen peroxide (H_2_O_2_), dinitrogen trioxide (N_2_O_3_) and peroxynitrite (ONOO^−^) are non-radical derivatives that may initiate free radical production [14].

Several external and internal stimuli in aerobic conditions lead to the production of low amounts of reactive species [15,16]. ROS are mainly generated by the mitochondrial electron transport chain (ETC) [17,18], where the electrons originating from the tricarboxylic acid (TCA) cycle are transferred; in the inner mitochondrial membrane, ubiquinone (coenzyme Q) transfers electrons originating from various suppliers (including Complex I and Complex II) to Complex III. The transfer of the first electron to Complex III triggers the temporary production of the free radical ubisemiquinone [18,19]; if the transfer of the second electron is delayed, ubisemiquinone can react with O_2_ at Complex IV, with consequent production of superoxide. Complex V is not involved in the electron transport, but a modification of its activity may alter the membrane potential, impacting ROS production [19].

A negligible percentage of O_2_ spent in physiological respiration is transformed to superoxide radical. The mitochondrial antioxidant system involves both enzymes, such as superoxide dismutases (SODs) and glutathione peroxidases (GPxs), and non-enzymatic scavengers, including glutathione (GSH, a co-factor/co-substrate of GPxs), normally allowing a limited amount of ROS production [14].

O_2_^.−^ can be metabolized to H_2_O_2_ by the copper/zinc superoxide dismutase (CuZnSOD, cytosolic, also known as SOD1) or the manganese superoxide dismutase (MnSOD, mitochondrial, also known as SOD2) [15]. O_2_·^−^ may be further scavenged by cytochrome c in the intermembrane space or may diffuse to the cytosol through the voltage-dependent anion channels (VDAC), which are pores located at the outer mitochondrial membrane (OMM) [18,20]. H_2_O_2_ is then detoxified by catalase (CAT), glutathione peroxidase (Gpx1) and peroxiredoxin (Prx). While GPx is the main defense against low amounts of reactive species, CAT achieves importance in severe oxidant production [15]. GPx1 and GPx4 isoforms are determinant in the scavenging of mitochondrial ROS. GPx1 is mostly cytosolic, but to a lesser extent, it is also located within the mitochondrial matrix, together with a specific mitochondrial GPx4 isoform. GSH-linked enzymes comprise mitochondrial and nuclear isoforms of glutaredoxin 2 (Grx2), thioredoxin 2 (Trx2) and thioredoxin reductase 2 (TrxR2) [16,18]. Glucose-6-phosphate dehydrogenase (G6PD) is another important enzyme for the modulation of redox homeostasis, since its main product, NAPDH, is required for the regeneration of GSH [21].

The production of reactive species is strictly controlled by scavenging systems. At low concentrations, reactive species generate mild oxidative stress and work as second messengers, activating or inhibiting different pathways, which include cell proliferation, apoptosis, metabolism modulation but also defense against microorganisms and immunity [22,23,24,25]. However, high amounts of reactive species induce high oxidative stress, with consequent metabolic changes and injury to biological macromolecules, such as DNA, lipids and proteins [15,16,20]. Reactive species may cause single- and double-stranded DNA breaks, contributing to premature aging, neurodegenerative diseases and cancer [20]. Lipid peroxidation impairs respiration, oxidative phosphorylation, membrane potential (Δψ) and calcium (Ca^2+^) buffering in mitochondria [20,26]. Oxidized proteins are identified and subsequently degraded by specific proteases [20,22]. A further consequence of oxidative stress is the initiation of cellular apoptosis or necrosis, triggered by both peroxidized lipids and mitochondrial Ca^2+^ excess, which stimulate the opening of the conductance cyclosporine A-sensitive permeability transition pore (PTP), with resulting depletion of cytochrome c and loss of ATP [27].

### 2.2. Redox Signaling: Modulation of Transcription Factors

Reactive species may modulate or may be modulated by several specific proteins, which include the nuclear factor kappa-light-chain-enhancer of activated B cells (NK-κB), the nuclear factor (erythroid-derived 2)-like 2 (NRF2), members of the Forkhead box O (FoxO) family, Wnt, the p53 (TRP53) tumor suppressor, the PR domain containing 16 (PRDM16) and Nucleoredoxin (Nrx) [28,29,30,31,32,33,34,35,36,37].

NF-κB is a master regulator in immune responses and inflammation, modulating the expression of several cytokines and other immune response genes [38]. Furthermore, NF-κB is determinant in other cellular processes, such as development, growth, survival and proliferation [39]. Cytosolic NF-κB activates and translocates to the nucleus in response to reactive species. Nevertheless, when NF-κB is oxidized, it cannot effectively bind DNA; the DNA-binding activity may be restored by reducing enzymes such as Trx [38].

NRF2 is a master modulator of redox biology, since it is implicated in the regulation of GSH and Trx levels, enzymes participating in phase I and phase II detoxification, NADPH regeneration and heme metabolism. NRF2 is further engaged in different cellular processes, including intermediary metabolism, autophagy, stem cell quiescence, innate and adaptive immunity [37,40]. NRF2 is sited in the cytosol and inhibited by the Kelch-like ECH-associated protein 1 (Keap1) forming a dimer; Keap1 further mediates the ubiquitination and degradation of NRF2 through the 26S proteasome. Reactive species modify two cysteines of Keap1, inducing its conformational modification and causing NRF2 dissociation and translocation to the nucleus, with subsequent binding to antioxidant response elements (AREs) [41,42,43,44].

Members of the FoxO family are involved in the control of cell metabolism, proliferation, resistance to stress, apoptosis and immune response [45]. Four FoxO isoforms were described in humans (1, 3, 4, 6), but many studies focused on FoxO1, FoxO3 and FoxO4 [36]. FoxOs modulate redox balance by upregulating antioxidant enzymes [36,46]. On the other hand, reactive species may regulate FoxO at numerous levels, including posttranslational modification, alterations in subcellular localization, interaction with coregulators, protein synthesis and stability [36].

Wnt proteins are required for basic developmental processes, including the control of cell-fate specification, stem cell proliferation and asymmetric cell division. Wnt proteins further regulate immune cell fate by modulating dendritic-cell maturation, regulatory T cell activation and effector T cell development [47]. Wnt proteins bind their receptor on the surface of target cells, promoting translocation of β-catenin within the nucleus and the transcription of downstream genes. β-catenin is stabilized by the cytoplasmic disheveled protein 1 (Dv1), which directly acts on downstream Wnt receptor (Frizzled). Dv1 binds to the Nucleoredoxin (Nrx), which is a sensor/effector of reactive species, rather than a scavenger. While Nrx increase promotes β-catenin degradation, its decrease induces Wnt-dependent gene expression [32]. Nrx can be oxidized and inactivated by NADPH oxidase 1 (Nox1)-generated ROS, dissociating from Dv1 and triggering the Wnt–β-catenin pathway [35].

Finally, reactive species are crucial for the inflammatory response by regulating the development and activation of the NOD-like receptor pyrin domain-containing 3 (NLRP3) inflammasome [48,49,50]. The inflammasome is a multiprotein complex involved in the cleavage and activation of caspase 1, which in turn induces the proteolytic cleavage of the premature form of IL-1β and IL-18 [51]. Even though the exact process through which reactive species mediate NLRP3 inflammasome activation and assembly needs to be fully elucidated, there are at least two proposed mechanisms. First, reactive species are sensed by a complex of Trx and Trx-interacting protein (TXNIP), inducing its dissociation and causing TXNIP to bind to the leucine-rich repeat of NLRP3, with final activation of NLRP3 [52]. A further mechanism is related to mtDNA binding; continuous production of reactive species may lead to mtDNA mutations, with consequent strand breaks and accumulation of free mtDNA in the cytoplasm, which may constantly activate NRLP3 inflammasome [53].

## 3. Immune Response in SARS-CoV-2 Infection

SARS-CoV-2 is mainly transmitted by exposure to microdroplets in the exhalates of infected individuals. Once inhaled, SARS-CoV-2 penetrates the bronchioles and alveoli where the main target respiratory epithelial cells are located. To infect the cells, SARS-CoV-2 binds to angiotensin-converting enzyme 2 (ACE2), which is considered as the principal viral entry receptor [54]. ACE2 expression has been reported on epithelial cells in the oral mucosa and alveoli, liver, kidney, intestine and heart [55,56]. Interestingly, ACE2 seems not to be expressed in immune system cells [57]. After cellular entry, SARS-CoV-2 activates both innate and adaptive immune responses, which have been characterized by several studies.

### 3.1. Innate Immune Response

Once SARS-CoV-2 enters the target cell, it is detected by pattern recognition sensor toll-like receptors (TLR) 3, 7, 8 and 9, as well as essential viral recognition receptor melanoma differentiation-associated protein 5 (MDA5) and retinoic acid-inducible gene-I (RIG-I) [58,59]. After ligand binding, recognition receptors activate crucial downstream transcription factors, including interferon regulatory factor (IRF), NF-κB and AP-1 [60]. SARS-CoV-2 detection activates the type 1 interferon (IFN) response program, with consequent induction of IFN-dependent gene expression [61].

The response mediated by TLR3 induces transcription of the NLRP3 gene, contributing to the activation of the NLRP3 inflammasome and consequent pyroptotic cell death [62]. The extent of inflammasome activation is associated with COVID-19 severity [63]. Release of the enzyme lactate dehydrogenase (LDH) occurs as a consequence of pyroptotic cell death. Increased circulating LDH levels have been detected and correlated with disease severity in patients affected by COVID-19 [64]. Through the extracellular delivery of gasdermin D, the NLRP3 inflammasome may also promote coagulopathy and severe thrombotic events, which characterize severe COVID-19 [65,66].

SARS-CoV-2 is able to stop nuclear translocation of signal transducers and activators of transcription (STATs) 1 and 2, with consequent transcriptional inhibition of IFN-stimulated genes [67]. The suppression of early type I IFN-mediated defense eases virus replication but causes the imbalance of immune response; indeed, it enhances a huge release of pro-inflammatory cytokines as a tentative to limit viral diffusion and to manage infected cells, causing considerable tissue injury and underlying a severe course of disease [68,69,70,71]. A comparison of the transcriptional response between other viruses shows that SARS-CoV-2 induces a particular signature characterized by reduced IFN-I and IFN-III responses, with significant induction of pro-inflammatory chemokines, such as IL-1β, IL-6, TNF and IL1RA [69].

Among the pro-inflammatory cytokines involved in innate immune response against SARS-CoV-2, IL-6 has been identified as a determinant pathogenic factor for the initiation of the acute respiratory distress syndrome in COVID-19 patients; it is thus considered a therapeutic target [72]. Increased circulating IL-6 levels may contribute to the activation of the complement system, since this cytokine is a strong inducer of the complement reactive protein (CRP). Indeed, circulating levels of the C5a complement factor are increased in proportion to the seriousness of COVID-19, and high expression levels of C5aR1 receptors are described in myeloid cells, supporting a role for the C5a–C5aR1 axis in the pathophysiology of severe COVID-19 [73]. Small-scale clinical studies have shown that treatment with complement modulators could be of benefit in COVID-19 patients, perhaps breaking off the complement-mediated activation of the coagulation system [74,75,76]. 

### 3.2. Adaptive Immune Response

Clinical evolution of SARS-CoV-2 infection relies on the switch between innate and adaptive immune response. This transition is determinant for the progress toward a protective immune response or an exacerbated inflammation [77,78]. SARS-CoV-2 infection induces an adaptive immune response, which is not dissimilar from other analogous viral infections, leading to the production of specific antibodies and consequent seroconversion occurring a few weeks after initial exposure [79,80]. After 7–10 days from the initial SARS-CoV-2 exposure, early antibody response is characterized by production and secretion of specific IgM and IgA [81,82,83]. The production of anti-spike IgG antibodies by B cells confers protection in exposed subjects [84]. IgG levels peak at approximately 50–60 days post-exposure and may last up to 10 months, but it is unknown whether their disappearance removes specific memory of the virus [85]. It is worth noting that powerful IgG response to SARS-CoV-2 may contribute to severe cytokine release syndrome and may be associated with increased disease severity and risk of death [78,86].

Circulating SARS-CoV-2-specific CD4+ and CD8+ T cells have been detected in COVID-19 convalescent patients [87]. In particular, CD4+ T cell response to the spike was strong and correlated with the extent of anti-SARS-CoV-2 IgG and IgA titers [87]. In patients with COVID-19, most immune cell types exhibited a powerful IFN-α response; intensive expansion of highly cytotoxic effector T cell subsets was associated with convalescence in moderate disease, while unbalanced IFN response, deep immune collapse with skewed T cell receptor repertoire and wide T cell expansion were described in severe disease [88]. A comprehensive mapping showed that functional CD4+ and CD8+ T cells, targeting multiple regions of SARS-CoV-2, are preserved in the resolution phase of both mild and severe COVID-19, and their magnitude correlates with the antibody response [89].

Interestingly, SARS-CoV-2-reactive CD4 T cells have been further detected in unexposed individuals, suggesting cross-reactive T cell recognition between circulating “common cold” coronaviruses and SARS-CoV-2 [87]. Pre-existing, cross-reactive immune memory of SARS-CoV-2 can be a determinant of COVID-19 severity [90]. Unexposed subjects were reported to present not only T cell reactivity, but also specific IgG anti-SARS-CoV-2 spike protein, even though their protective efficacy against COVID-19 is still controversial [91,92].

Previous exposure to other coronaviruses may further elicit the antibody-dependent enhancement (ADE), which occurs when antibodies are not able to neutralize the virus but ease its cellular entry and replication, further maintaining inflammation and cytokine storm [93]. ADE has been reported for SARS-CoV in a hamster model and in human promyelocytic cells, as well as in patients affected by SARS. The interaction with Fc receptors of anti-SARS-CoV antibodies complexed with virions enhanced viral cell entry and replication and a modulated pro-inflammatory cytokine response [94,95,96]. Nonetheless, clear evidence of ADE in SARS-CoV-2 infection has not yet been reported.

## 4. COVID-19, Redox Balance and Immunity

### 4.1. COVID-19 Is Characterized by Impaired Redox Homeostasis

Due to its crucial role in response to infections, oxidative stress is considered a key determinant in COVID-19 pathogenesis [97,98]. Pathological changes underlying pulmonary damage induced by SARS-CoV-2 include exudative proteinaceous injury and inflammatory lymphocytic infiltrates, diffuse alveolar damage with hyaline membranes and wall thickening [99]. Severe COVID-19 is further characterized by hypercoagulation and hypoxia in several organs [100]. Such massive induction of tissue damage can be related to a defective redox balance [101].

Excess of reactive species and consequent dysregulated redox homeostasis were described in several respiratory viral infections. Following activation of innate immunity and pro-inflammatory cytokines, infection by respiratory syncytial virus induces overproduction of reactive species, increasing lipid peroxidation, depleting GSH and inhibiting NRF2 in respiratory epithelial cells [102,103]. Influenza virus leads to reactive species excess in several tissues, particularly in lungs, inducing apoptosis and cytotoxicity, but activating NRF2 to counteract oxidative injury in alveolar epithelial cells [104,105,106]. Several pre-clinical studies suggest that severe lung damage in SARS-CoV infection relies on both oxidative stress and innate immunity, with consequent activation of NF-κB and enhanced pro-inflammatory host response [107,108,109]. In convalescent patients, an upregulation of mitochondrial and redox-sensitive genes occurs, supporting the association between redox imbalance, inflammation and the pathogenesis of SARS-CoV infection [110].

According to previous evidence from similar viral infections, impairment in redox balance may deeply impact COVID-19 pathogenesis, even though reports sustaining this hypothesis are still limited. Several radical scavengers, such as GSH, NADPH or Trx, may regulate the cellular disulfide–thiol balance, which is crucial for SARS-CoV-2 entry and fusion into the host cell [97]. Deficiency of G6PD may be associated with severe COVID-19, since redox homeostasis mediated by this enzyme is involved in the immune response to viral infections [21]. This is strongly suggested by the following evidence: (1) G6PD deficiency enhances several viral infections; (2) G6PD variants may impact COVID-19 severity; and (3) higher incidence of COVID-19 in African-Americans, whose G6PD deficiency is characterized by higher oxidative stress [111,112,113,114]. A pilot study on COVID-19 patients hospitalized in intensive care unit showed reduced circulating levels of several antioxidants (such as vitamin C, thiol proteins, GSH, γ-tocopherol, β-carotene), as well increased lipid peroxides and the oxidative stress index copper/zinc ratio [115]. When the total oxidant status, total antioxidant capacity and level of glutathione were compared in hospitalized COVID-19 patients with different disease severity, increased oxidative stress indices and reduced antioxidant markers were related to serious clinical presentation and outcomes [116]. Nevertheless, another study showed no correlation observed between the oxidative stress parameters and the degree of COVID-19 severity in hospitalized patients, suggesting that disease severity may not contribute to redox changes in SARS-CoV-2 infection [117]. A preliminary report on a small sample of critically ill COVID-19 patients described higher levels of protein adducts of the lipid peroxidation product 4-hydroxynonenal in the deceased, as compared to survivors [118]. Compared to healthy controls, circulating SOD and CAT activity, as well as carbonyl and lipid peroxidation (LPO) levels, were higher, while total antioxidant capacity levels were lower in COVID-19 patients [119]. Moreover, higher LPO levels were independently associated with a higher risk of intubation or death at 28 days [119]. A further study demonstrated that neutrophils are the main source of reactive species in severe COVID-19, and circulating H_2_O_2_ levels are increased in dead patients [120].

### 4.2. Altered Redox Balance Modulates the Immune Response

**Figure 2:**
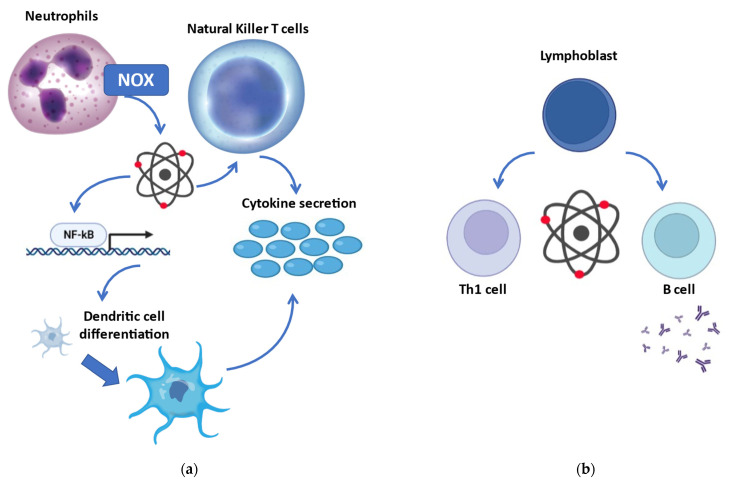
Redox regulation of the immune response. (**a**) Involvement of redox biology in innate immunity: more than being produced by the oxidative burst in phagocytes, reactive species may oxidize the transcription factor NF-κB with consequent activation of several cells engaged in the innate immune response. (**b**) Redox modulation of adaptive immunity: reactive species are implicated in the differentiation of secretive B cells and Th1 cells (while reducing compounds promote Th2 cells). NOX, NADPH oxidase; NF-κB, nuclear factor kappa-light-chain-enhancer of activated B cells.

Redox biology accomplishes key regulatory functions in innate immunity (Figure 2a). Increased production of reactive species by phagocytes is one of the first-line antimicrobial responses, defined as the oxidative/respiratory burst [121]. More than merely producing reactive species via NADPH oxidase, neutrophils may sense the differential localization of oxidants and finely tune IL-1β expression through selective oxidation of NF-κB [122]. Mitochondria-derived reactive species further regulate the differentiation process of dendritic cells [123]. Furthermore, mitochondrial reactive species in macrophages enter the cytosol and induce a covalent modification in NF-κB essential modulator (NEMO), an element of the inhibitor of κB kinase (IKK) complex required to activate both the ERK1/2 and NF-κB pathways and to promote secretion of pro-inflammatory cytokines [124]. Reactive species are also produced by NADPH oxidase in natural killer T cells (but not in CD4+ or CD8+ T cells), modulating their expression of IFN-γ and IL-17, thus playing a role in the regulation of inflammatory function [125]. 

Redox signaling and regulation are crucial in adaptive immunity (Figure 2b). Indeed, excessive production of reactive species is associated with the activation and differentiation of both T and B cells. T helper activation, required for both humoral and cell-mediated immune response, relies on the redox status of the microenvironment [126]. Even though a reduced microenvironment could protect from oxidative stress during T cell activation, mild concentrations of reactive species are necessary for the initiation of adaptive immune response [127]. Activation of CD28, costimulatory of T cell activation, induces intracellular reactive species, with consequent induction of IL-2 via NF-κB [128]. On the other hand, the antioxidant GSH is required to regulate the proliferation of activated T cells [129]. Nevertheless, low amounts of reactive species are a precondition for T cell survival, while oxidant accumulation causes apoptosis/necrosis [130]. Moreover, redox signaling may affect T cell commitment, and different T cells present with various redox levels [130]. Indeed, oxidative status induces Th1 development, while prevalence of reducing molecules shifts toward Th2 responses [131].

## 5. Targeting Impaired Redox Homeostasis in COVID-19

Several studies suggest that redox balance may be a feasible therapeutic target for COVID-19 by modulating the redox-sensitive immune response. Numerous trials have been designed to test antioxidants (such as N-acetylcysteine, ascorbic acid, resveratrol) in COVID-19, and many of them are currently ongoing while this review is being written. 

N-acetylcysteine (NAC) could potentially treat COVID-19 infection by stimulating glutathione synthesis, promoting T cell response and regulating inflammation [132]. Other than providing an extra source of cysteine to synthetize glutathione, NAC can stop ACE2 activity and SARS-CoV-2 entry into target cells by the presence of a thiol group [133]. NAC was intravenously administered in patients with severe COVID-19, contributing to clinical improvement, as well as reduction of C-reactive protein [134]. A retrospective two-center cohort study showed that oral NAC reduces the risk of mechanical ventilation and mortality when added to standard of care in patients with moderate to severe COVID-19 [135]. However, a double-blind randomized trial was not able to demonstrate that intravenous administration of NAC in high doses was superior to placebo in affecting the evolution of severe COVID-19 [136]. Similar results were observed in a pilot study, which could not support beneficial effects of intravenous NAC in COVID-19 patients with acute respiratory distress syndrome [137].

Ascorbic acid (vitamin C) is a potent antioxidant, which directly scavenges reactive species, and it is also highly concentrated in leukocytes for several immune responses [138,139]. The first suggestion on the efficacy of vitamin C in reducing susceptibility to respiratory tract infections derives from Linus Pauling [140]. Circulating levels of ascorbic acid were severely depleted in COVID-19 patients with acute respiratory distress syndrome [141]. Administration of high-dose intravenous vitamin C was associated with improved inflammatory and immune response, as well as restored organ function in severe/critical COVID-19 [142]. Nevertheless, a pilot study failed to demonstrate any clinical improvement in critically ill COVID-19 patients treated with intravenous high doses of ascorbic acid [143]. Two randomized controlled trials could not demonstrate that the addition of intravenous vitamin C to standard therapy had an impact on mortality, length of stay or the need for mechanical ventilation in COVID-19 patients [144,145].

Resveratrol is a polyphenol with several antioxidant, anti-inflammatory and immunomodulator properties [146]. More than being a simple scavenger of reactive species, resveratrol increases the expression of the antioxidant protein SIRT1, which in turn boosts NAD levels, improving the immune response [147]. Resveratrol has been demonstrated to reduce the replication of SARS-CoV-2 in vitro, showing antiviral properties in infected human bronchial epithelial cells [148,149]. In a placebo-controlled cross-over study, resveratrol supplementation in obese men reduced the expression of ACE2 in adipose tissue, suggesting that this compound could reduce SARS-CoV-2 diffusion [150]. A randomized placebo-controlled phase 2 trial showed that oral supplementation of resveratrol in COVID-19 outpatients presented with lower incidence of pneumonia and hospitalization [151]. 

Pharmacological activation of the transcriptional factor NRF2 has been recently suggested as a promising therapeutic strategy against COVID-19 due to restoration of redox homeostasis and resolution of inflammation [152]. Sulforaphane is an electrophile that modifies cysteine sensors of Keap1, inactivating its repressor functions [153]. This compound is able to inhibit the replication of SARS-CoV-2 in vitro and in the upper respiratory tract or lungs of SARS-CoV-2-infected mice, reducing pulmonary injury [154]. Furthermore, sulforaphane inhibits the expression of IL-6 and IL-8 in cultured bronchial cells exposed to the S-protein of SARS-CoV-2, supporting its anti-inflammatory effect [155]. Nevertheless, no clinical data on the efficacy of sulforaphane are currently available. Bardoxolone and bardoxolone methyl are electrophilic moieties able to activate the NRF2 pathway and inhibit the NF-κB pathway [156]. Both compounds can inhibit SARS-CoV-2 replication by specifically binding the 3C-like protease in infected Vero cells [157]. Hence, even these Nrf2 activators may be considered in a multifaceted antiviral treatment strategy.

Other compounds involved in the interplay with redox homeostasis were suggested to be potentially beneficial in the treatment of COVID-19. Polyphenols are natural agents with high antioxidant and anti-inflammatory properties, which could also target virus proteins or cell receptors, preventing SARS-CoV-2 entry and replication [158]. Acting as anti-inflammatory and antioxidant, the bioactive molecule melatonin may be effective in reducing acute lung injury caused by SARS-CoV-2 [159]. The trace element zinc—whose deficiency is reported in severe COVID-19—may prevent SARS-CoV-2 by improving respiratory tissue barrier and inhibiting viral replication, but also balancing immune response and redox homeostasis [160]. The novel antifibrotic agent pirfenidone could reduce inflammation and counteract oxidative stress, antagonizing apoptosis and downregulating ACE2 expression [161]. Selenium is another trace element incorporated in several selenoproteins with both anti-inflammatory and antioxidant functions; the expression of several selenoproteins is decreased by SARS-CoV-2 infection, and redox-active selenium molecules might potentially inhibit SARS-CoV-2 proteases [162]. All these redox compounds can be considered as promising in counteracting SARS-CoV-2 infection and modulating an immune response to COVID-19, even though further studies are needed.

## 6. Conclusions

Literature evidence strongly supports impairment of redox balance as the main determinant of SARS-CoV-2 infection and COVID-19 pathogenesis, contributing to cytokine storm, immune dysregulation and intravascular coagulation mechanisms, thus promoting disease severity. Knowledge progression of several redox-dependent pathways controlling the immune response in SARS-CoV-2 infection is currently ongoing, suggesting an intricate interplay between the loss of redox homeostasis and cytokine storm as a mechanism that amplifies tissue injury, leading to organ failure. Clinical trials using redox modulators to counteract COVID-19 are progressing, but additional research is required to identify and test further specific therapeutic targets, leading to the definition of clinical practice guidelines for the early treatment of SARS-CoV-2 infection and full management of severe COVID-19.

## Data Availability

Not applicable.
